# LAPAROSCOPIC BARIATRIC SURGERY IN ADOLESCENTS: EARLY AND FIVE- YEAR CLINICAL AND LABORATORY ASSESSMENT

**DOI:** 10.1590/0102-672020230030e1748

**Published:** 2023-07-17

**Authors:** Lorena Gottardi, Augusto Tinoco, Luiz Ronaldo Alberti

**Affiliations:** 1 Hospital São José do Avaí, Surgery Unit – Itaperuna (RJ), Brazil; 2 Santa Casa de Misericórdia – Belo Horizonte (BH), Brazil.

**Keywords:** Pediatric obesity, Adolescent health, Bariatric surgery, Comorbidity, Obesidade infantil, Saúde do adolescente, Cirurgia bariátrica, Comorbidade

## Abstract

**BACKGROUND:**

Obesity has reached epidemic proportions among adolescents. Methods, such as bariatric surgery, have become the most effective treatment for patients with classes III and IV obesity.

**AIM:**

To evaluate weight loss, comorbidity remission, and long-term results of bariatric surgery in adolescents.

**METHODS:**

Study with adolescent patients undergoing bariatric surgery, evaluating laboratory tests, comorbidities, and the percentage of excess weight loss in the preoperative period and at one, two, and five years postoperatively.

**RESULTS:**

A total of 65 patients who met the inclusion criteria, with a mean age of 18.6 years, were included in the analysis. In the preoperative period, 30.8% of hypercholesterolemia, 23.1% of systemic arterial hypertension, and 18.4% of type 2 diabetes were recorded, with remission of these percentages occurring in 60, 66.7 and 83.4%, respectively. The mean percentage of excess weight loss was 63.48% after one year of surgery, 64.75% after two years, and 57.28% after five years. The mean preoperative total cholesterol level was 180.26 mg/dL, and after one, two, and five years, it was 156.89 mg/dL, 161.39 mg/dL, and 150.97 mg/dL, respectively. The initial mean of low-density lipoprotein was 102.19mg/dL and after five years the mean value reduced to 81.81 mg/dL. The mean preoperative glycemia was 85.08 mg/dL and reduced to 79.13 mg/dL after one year, and to 76.19 mg/dL after five years.

**CONCLUSIONS:**

Bariatric surgery is safe and effective in adolescents, with low morbidity, resulting in a loss of excess weight and long-term stability, improving laboratory tests, and leading to remission of comorbidities, such as diabetes mellitus, hypercholesterolemia, and systemic arterial hypertension.

## RESUMO

**RACIONAL:**

A obesidade tomou proporções epidêmicas entre adolescentes, e procedimentos como a cirurgia bariátrica tornou-se o tratamento mais efetivo em pacientes com obesidade grau III e IV.

**OBJETIVOS:**

Avaliar a perda peso, a remissão de comorbidades, e resultados a longo prazo da cirurgia bariátrica em adolescentes.

**MÉTODOS:**

Estudo com pacientes adolescentes submetidos à cirurgia bariátrica, avaliando exames laboratoriais, comorbidades e o percentual de perda de excesso de peso, nos períodos pré-operatório e com 1, 2 e 5 anos de pós-operatório.

**RESULTADOS:**

Foram incluídos no estudo 65 pacientes que preencheram os critérios de inclusão, com a média de idade de 18,6 anos. No pré-operatório foram registrados: 30,8% hipercolesterolemia, 23,1% hipertensão arterial sistêmica e 18,4% diabetes tipo 2, ocorrendo remissão destes porcentuais em 60, 66,7 e 83,4%, respectivamente. O percentual médio de perda de excesso de peso após 1 ano foi de 63,48%, após 2 anos foi de 64,75% e após 5 anos foi 57,28%. O valor médio do colesterol total no pré-operatório era de 180,26mg/dL, e após 1 ano, 2 anos e 5 anos foram de 156,89mg/dL,161,39mg/dL e de 150,97mg/dL, respectivamente. A média inicial de lipoproteína de baixa densidade era 102,19mg/dL e após 5 anos o valor médio reduziu para 81,81 mg/dL. O valor médio da glicose pré-operatório era 85,08 mg/dL, após um ano uma média 79,13mg/dL, e com 5 anos 76,19 mg/dL.

**CONCLUSÕES:**

A cirurgia bariátrica é segura e eficaz em adolescentes, com baixa morbidade, resultando em uma perda do excesso de peso e estabilidade a longo prazo, melhorando exames laboratoriais e levando a remissão de comorbidades como diabetes mellitus, hipercolesterolemia e hipertensão arterial sistêmica.

## INTRODUCTION

Obesity is a chronic disease characterized by the excessive accumulation of adipose tissue, which is one of the main nutritional changes with pathological complications in adolescents^13^.

The World Health Organization (WHO, 2013) estimates that approximately 42 million children under five years of age have excess weight, most of them living in countries with middle to high socioeconomic development^27^. According to the Brazilian Institute of Geography and Statistics (IBGE), obesity prevalence in 2016 was 124 million children and adolescents from 5 to 19 years of age worldwide^18^.

In accordance with the classification of obesity by body mass index (BMI), which is calculated using the formula weight/height^2^, adults are considered obese when BMI >30 kg/m^2^. In adolescents, however, BMI must be used in association with the WHO growth curves (2007), which are sex- and age-specific and consider percentiles over 85 and 95 or Z-scores +2 and +3 to determine a diagnosis of overweight and obesity, respectively^14,28^.

The selection criteria for bariatric surgery in adolescents include BMI >40 kg/m^223^, and a BMI of 35 kg/m^2^ or above associated with major comorbidities (such as type 2 diabetes, moderate or severe sleep apnea, severe fatty liver disease, systemic arterial hypertension [SAH], and hypercholesterolemia).

There are few systematic reviews on the long-term results of bariatric surgery, comorbidity remission, and weight loss in adolescents^6^. Bariatric surgery has been established as an effective treatment for severe obesity in adolescents^7^. Studies have demonstrated that early surgical intervention leads to the disappearance of existing comorbidities, such as type 2 diabetes and SAH^1^.

In studies of patients submitted to laparoscopic Roux-en-Y gastric bypass (RYGB) and sleeve gastrectomy (SG), it was observed that RYGB resulted in a more lasting weight loss in adolescents than SG, with complication rates similar to those observed in adults^17^.

The present study aims to evaluate pre- and postoperative (one, two, and five years) clinical and laboratory parameters in adolescents submitted to laparoscopic bariatric surgery and assess the long-term maintenance of weight loss and the improvement of these patients’ comorbidities.

## METHODS

This is a retrospective study involving patients who underwent laparoscopic SG and RYGB between 2010 and 2018 at a Brazilian community hospital. The hospital had a multidisciplinary team, including surgeons, endocrinologists, nutritionists, psychologists, and physical therapists, which allowed patients who undergo the procedure to have their treatment adequately followed-up.

The inclusion criteria were patients aged between 14 and 20 years, of both sexes, who were evaluated by the multidisciplinary team, and underwent SG or RYGB; with stable weight, defined as no changes >3.0% in the three months before their inclusion; possibility of clinical follow-up through their medical records for one, two, and five years; patients with BMI >40 kg/m^2^ or with BMI >35 kg/m^2^ accompanied by comorbidities in accordance with the indications for surgery of the Brazilian Society of Bariatric and Metabolic Surgery; a signed informed consent form relative to the choice of the type of surgery and any research derived thereof, which was read and explained, and any concerns were clarified in a consultation with the surgeon. The study was approved by the Ethics Committee of the Institution under 31709320.6.0000.5138.

The following tests were performed and analyzed one to three months before bariatric surgery and at one, two, and five years after surgery: fasting glycemia, total cholesterol, high-density lipoprotein (HDL), low-density lipoprotein (LDL), and triglycerides. In addition, the percentage of excess weight loss (%EWL) was calculated as: (preoperative weight – current weight) ×100/ (preoperative weight – ideal weight). The surgery was considered a failure when %EWL <50% in a period shorter than two years.

**Figure 1:**
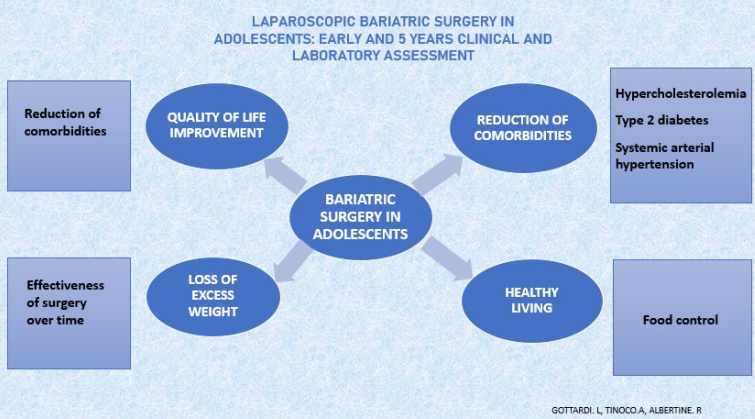
Advantages of performing bariatric surgery in severe obesity in adolescents.

Figure 1 summarizes the advantages of performing bariatric surgery in severe obesity in adolescents.

## RESULTS

A total of 96 patients underwent bariatric surgery from 2010 to 2018; however, due to lack of follow-up, only 65 patients were included in the sample. Of these, 65% were submitted to SG and 35% to RYGB, of whom 80% were women. The mean age was 18.6 years (standard deviation [SD]±1.35), with a minimum age of 14 years and a maximum, of 20 years. The most frequent comorbidity was gastroesophageal reflux disease (GERD), present in 36.9% of the sample, followed by sleep apnea and hypercholesterolemia (30.8%), SAH (23.1%), and type 2 diabetes mellitus (18.4%).

The statistical analysis of the comparisons between preoperative values and values at one, two, and five years after surgery showed that there were significant differences in all the evaluated parameters, with borderline significance for HDL (Table 1).

**Table 1:** Comparison of the patients’ total cholesterol, high-density lipoprotein, low-density lipoprotein, triglycerides, and glycemia over time in the period from 2010 to 2018(*).

	Pre-surgery	after 1 year	after 2 years	after 5 years	p-value
Cholesterol	180,26 (37,87)	156,89 (27,50)	161,39 (38,82)	150,97(31,56)	0,025
Triglycerides	128,06 (100,68)	86,96 (38,40)	86,60 (33,60)	81,37 (30,59)	0,038
HDL	53,18 (15,57)	55,51 (14,55)	65,13 (21,70)	56,27 (11,53)	0,051
LDL	102,19 (32,93)	87,19 (25,82)	82,62 (25,61)	81,81 (20,41)	0,028
Glycemia	85,08 (11,71)	79,13 (8,23)	80,50 (8,05)	76,19 (15,72)	0,012

HDL: high-density lipoprotein; HDL: low-density lipoprotein.

* ANOVA Analysis of Variance; Results expressed as means (±SD).

The mean cholesterol level before surgery was 180.26 mg/dL and reduced to 156.89 mg/dL after one year, to 161.39 mg/dL after two years, and to 150.97 mg/dL (p-value [p]=0.025) after five-year follow-up.

A significant decrease in triglyceride levels was also observed over the study period. The baseline mean value was 128.6 mg/dL and later reduced to 86.26 mg/dL after one year, and at the end of follow-up the mean level was 81.37 mg/dL (p=0.038). LDL decreased significantly over time, with a mean baseline level of 102.19 mg/dL reducing to 81.81 mg/dL after five years (p=0.028).

The mean preoperative glycemia was 85.08 mg/dL; after one and five years of follow-up, it was 79.13 mg/dL and 76.19 mg/dL (p=0.012), respectively (Table 2).

**Table 2:** Multiple comparisons of total cholesterol, high-density lipoprotein, low-density lipoprotein, triglycerides, and glycemia in the evaluated follow-up periods for patients operated from 2010 to 2018.

Multiple comparisons**						
	Preoperative vs. 1 year	Preoperative vs. 2 years	Preoperative vs. 5 years	1 year vs. 2 years	1 year vs. 5 years	2 years vs. 5 years
Cholesterol	0.000	0.008	0.001	0.368	0.700	0.379
Triglycerides	0.001	0.012	0.003	0.304	0.501	0.360
HDL	0.362	0.001	0.808	0.053	0.906	0.283
LDL	0.002	0.000	0.001	0.383	0.662	0.644
Glycemia	0.000	0.127	0.013	0.005	0.242	0.490

** Paired t test.

HDL: high-density lipoprotein; HDL: low-density lipoprotein.

All parameters, with the exception of HDL, significantly improved from their preoperative values to the 1st, 2nd, and 5th years after surgery. However, the results were not statistically significant when comparing total cholesterol, triglyceride, and LDL levels after the 1st year of follow-up with the other periods (Table 2).

Remission of comorbidities occurred in 83.4% of the patients with type 2 diabetes mellitus, in 66.7% of those with SAH, in 60% of those with hypercholesterolemia, and in 70% of those with apnea.

The mean %EWL one year after surgery was 63.48%. The mean %EWL was 64.75% at two years after surgery (range 18–99%), and 57.28% after five years (range 14–93%), which was a significant weight loss, proving the long-term effectiveness of bariatric surgery due to weight loss maintenance. The analysis of %EWL by sex showed that men had greater weight loss (75.23%) than women (60.50%) in the first year of follow-up (p=0.036; Table 3).

**Table 3:** Percent excess weight (%) by sex at one, two, and five years after surgery.

Percent excess weight (%)		n	Mean	Standard deviation	Mean difference	Confidence interval (%)	p-value*
1 year	Woman	51	60,50	23,53	−14,73	−28,44– −1,02	**0,036**
	Men	13	75,23	14,59			
2 years	Woman	27	61,67	20,55	−19,73	−39,66–0,19	0,052
	Men	5	81,40	16,36			
5 years	Woman	24	54,21	24,18	−21,54	−47,34–4,25	0,098
	Men	4	75,75	14,03			

* *t*-test.

The percentage of patients who regained weight was 15.4%. In the sample, 20% failed to lose weight, meaning that they did not reach the cutoff level of 50% weight loss required to demonstrate the effectiveness of surgery. Of these 20, 4.6% converted from SG to RYGB over two years. After the second surgery, these patients were able to reach the desired weight loss and kept their %EWL above 50%, which demonstrated the effectiveness of the surgery. Only one patient underwent conversion from SG to RYGB due to GERD.

The evaluation of immediate postoperative complications showed that 1.5% of patients presented with a fistula 10 days after RYGB surgery. During the study period, 12.3% also underwent a videolaparoscopic cholecystectomy. Some metabolic complications were observed, such as anemia in 13.8% of the patients, calcium deficiency in 44.6 %, vitamin D deficiency in 24.6%, and vitamin B^12^ deficiency in 20%. No deaths were recorded in this study.

## DISCUSSION

Bariatric surgery is a safe and effective treatment for adolescents as evidenced by this study.

The follow-up of treatment must be conducted in accordance with protocols to ensure that weight loss and reduction of the comorbidities caused by obesity are maintained. As the harmful consequences of obesity are postponed or delayed, it is possible to enable an improvement in quality of life and a change in habits^21,24^.

Oberbach et al. concluded that obese adolescents show a considerable improvement in their metabolic comorbidities after surgical obesity treatment^19^. Al-Qahtani et al. described a reduction of comorbidities in their data, including dyslipidemia by 70.0%, SAH by 75.0%, pre-SAH by 83.3%, symptoms of obstructive sleep apnea by 90.9%, diabetes by 93.8%, and prediabetes by 100.0%^2^. The same were observed in the present study, in which all studied comorbidities decreased, including hypercholesterolemia with reduction persisting after five years in 60% of patients, five-year reduction of mean total cholesterol from 180.26 mg/dL to 150.97 mg/dL, and mean triglycerides decrease from 128.06 mg/dL to 81.37 mg/dL in the corresponding period.

Armstrong et al. stated that bariatric surgery shows significant and sustained antidiabetic effects in adolescents, similar to those found in adults^4^. A review of type 2 diabetes mellitus remission rates, in long-term prospective multicenter studies, with 35 diabetic adolescents showed that approximately 89% achieved remission five years after bariatric surgery^20^. This was also observed in the present study, in which the mean glycemia in type 2 diabetes mellitus cases was reduced from 85.08 mg/dL before surgery to 83.33 mg/dL, which further decreased to 76.19 mg/dL after five years of follow-up, corroborating the aforementioned studies.

The most widely used standards to assess surgery effectiveness are %EWL and weight loss maintenance. In many studies, it was possible to perform this assessment based on %EWL in the first two years, as in Van de Laar et al. (2018), who reported a total of 51 patients aged under 19 years who underwent SG and had a %EWL of 94.6, 96.2, and 92.9% at six months, one year, and two years, respectively^26^. Al-Qahtani et al. analyzed 108 adolescents with a mean baseline BMI of 50 kg/m^2^ who were followed for up to two years and obtained a mean %EWL of 62%^12^. In the present study, a significant reduction in excess weight was observed, with an additional long-term follow-up of five years after surgery, and the mean %EWL was 63.48%, 64.75%, and 57.28% after one, two, and five years, respectively. These results validate surgery's long-term effectiveness.

In the Khidir et al. work, a difference in %EWL was found between male and female adolescents: 51±25.39 and 46±25.04, respectively^13^. A likewise trend was observed in the present analysis, with male patients presenting a higher loss (75.23%) in the first year of follow-up than female patients, who exhibited a %EWL of 60.50% in the corresponding period. The same pattern of weight loss was observed in adults.

In 2017, a multicenter study of adolescents, who underwent bariatric surgery, showed an increase in weight in two years compared to the first year after surgery^5^. In contrast, Khidir et al. showed that the weight loss persisted in their study group up to five years after surgery, with a total weight loss percentage of 35.8±11.5%, therefore indicating a low weight regain rate. The same was observed in the present sample, with a 15.4% weight regain rate after five years and a persistent 65% %EWL in the corresponding period.

Adherence to instructions of the medical team and to multidisciplinary follow-up is essential for a successful treatment after bariatric surgery in pediatric and adolescent patients, as shown in a meta-analysis with 73 patients, of which 28.7% did not return or undergo any evaluation follow-up in two years^16^. In the present study, 31 patients (32.2%) were excluded due to the absence of feedback after surgery, personally or by telephone, similarly to the aforementioned study.

Bariatric surgery is associated with a low complication rate, regardless of the type of procedure and the analyzed group. These surgical procedures are generally well tolerated, with a minimal risk of serious complications during surgery^8,10,20^. One article reported a total rate of immediate postoperative complications (<30 days) of 2.4% in patients aged 18 to 21 years^3^. In the present study, the rate of immediate postoperative complications was 1.5%, with all cases resolved without further repercussions to the patient and with no interference in the effectiveness of the surgery, once the weight loss persisted continuously over the analyzed period. During follow-up, 12.3% of patients underwent videolaparoscopic cholecystectomy. It must be emphasized that no deaths were recorded in this study.

Some long-term complications stood out, particularly micronutrient deficiencies, such as calcium, 25-hydroxyvitamin D, iron, and vitamins B^1^, B^6^, B^12^, and A, which without supplementation or with inadequate absorption can result in serious nutritional complications^29^. Thus, it is essential to conduct a follow-up that detects any micro- and macronutrient alterations at an early stage, thereby minimizing the development of such deficiencies and their complications^22^.

In the AMOS (Adolescent Morbid Obesity Surgery) study by Olbers (2017), 72% of patients presented some sort of nutritional deficiency. On the other hand, although vitamin deficiency was reported in the FABS-5+ (Follow-up of Adolescent Bariatric Surgery at 5 Plus Years) study by Inge et al., it was not quantified. In both studies, the repercussions were mild and controlled by supplementation^11^.

In the present study, calcium, vitamin D, and vitamin B^12^ deficiencies were identified in 44.6, 24.6, and 20% of the adolescents, respectively. All patients remained under supplementation and there were no effects from those deficiencies.

Postoperative anemia was found in 46% of patients in the FABS-5+ study by Inge et al. and in 33% of the patients in the AMOS study by Olbers^11,20^. Anemia due to iron deficiency and vitamin B^12^ deficiency after gastric bypass is described in many studies^12,15^. In the present study, 13.2% of the adolescents had anemia, which was corrected with medication.

Some authors reported a high reoperation rate due to surgery failure in adolescents submitted to SG^9^, unlike the present study, in which the conversion rate from SG to RYGB was 4.6% (three cases, of which two were due to surgery failure for not reaching a %EWL of 50%, and one was due to GERD).

The reported data confirm the viability of bariatric surgery, the maintenance of clinically significant weight loss, the improvement of the main comorbidities, and, consequently, of weight-related quality of life among adolescents submitted to surgery as a form of treatment for obesity^25^.

## CONCLUSIONS

Bariatric surgery in adolescents is safe and effective. It can be performed with low morbidity and results in stable long-term weight loss. In addition, it improves laboratory test results, leading to a remission of comorbidities, such as diabetes mellitus, hypercholesterolemia, and SAH.

## References

[1] Allen SR, Lawson L, Garcia V, Inge TH (2005). Attitudes of bariatric surgeons concerning adolescent bariatric surgery (ABS). Obes Surg.

[2] Alqahtani AR, Antonisamy B, Alamri H, Elahmedi M, Zimmerman VA (2012). Laparoscopic sleeve gastrectomy in 108 obese children and adolescents aged 5 to 21 years. Ann Surg.

[3] Altieri MS, Pryor A, Bates A, Docimo S, Talamini M, Spaniolas K (2018). Bariatric procedures in adolescents are safe in accredited centers. Surg Obes Relat Dis.

[4] Armstrong SC, Bolling CF, Michalsky MP, Reichard KW, SECTION ON OBESITY, SECTION ON SURGERY (2019). Pediatric metabolic and bariatric surgery: evidence, barriers, and best practices. Pediatrics.

[5] Benedix F, Poranzke O, Adolf D, Wolff S, Lippert H, Arend J (2017). Staple line leak after primary sleeve gastrectomy-risk factors and mid-term results: do patients still benefit from the weight loss procedure?. Obes Surg.

[6] Black JA, White B, Viner RM, Simmons RK (2013). Bariatric surgery for obese children and adolescents: a systematic review and meta-analysis. Obes Rev.

[7] Cozacov Y, Roy M, Moon S, Marin P, Lo Menzo E, Szomstein S (2014). Mid-term results of laparoscopic sleeve gastrectomy and Roux-en-Y gastric bypass in adolescent patients. Obes Surg.

[8] Furtado TA, Girundi MG, Campolina COC, Mafra SC, Oliveira AMO, Santos MLPDD (2023). Depressive and eating disorders in patients post-bariatric surgery with weight regain: a descriptive observational study. Arq Bras Cir Dig.

[9] Himpens J, Dobbeleir J, Peeters G (2010). Long-term results of laparoscopic sleeve gastrectomy for obesity. Ann Surg.

[10] Inge TH, Coley RY, Bazzano LA, Xanthakos SA, McTigue K, Arterburn D (2018). Comparative effectiveness of bariatric procedures among adolescents: the PCORnet bariatric study. Surg Obes Relat Dis.

[11] Inge TH, Jenkins TM, Xanthakos SA, Dixon JB, Daniels SR, Zeller MH (2017). Long-term outcomes of bariatric surgery in adolescents with severe obesity (FABS-5+): a prospective follow-up analysis. Lancet Diabetes Endocrinol.

[12] Karefylakis C, Näslund I, Edholm D, Sundbom M, Karlsson FA, Rask E (2015). Prevalence of anemia and related deficiencies 10 years after gastric bypass--a retrospective study. Obes Surg.

[13] Khidir N, El-Matbouly MA, Sargsyan D, Al-Kuwari M, Bashah M, Gagner M (2018). Five-year Outcomes of laparoscopic sleeve gastrectomy: a comparison between adults and adolescents. Obes Surg.

[14] Kimm SY, Barton BA, Obarzanek E, McMahon RP, Sabry ZI, Waclawiw MA (2001). Racial divergence in adiposity during adolescence: the NHLBI growth and health study. Pediatrics.

[15] Kotkiewicz A, Donaldson K, Dye C, Rogers AM, Mauger D, Kong L (2015). Anemia and the Need for Intravenous Iron Infusion after Roux-en-Y Gastric Bypass. Clin Med Insights Blood Disord.

[16] Lainas P, De Filippo G, Di Giuro G, Mikhael R, Bougneres P, Dagher I (2020). Laparoscopic sleeve gastrectomy for adolescents under 18 years old with severe obesity. Obes Surg.

[17] Lawson ML, Kirk S, Mitchell T, Chen MK, Loux TJ, Daniels SR (2006). One-year outcomes of Roux-en-Y gastric bypass for morbidly obese adolescents: a multicenter study from the Pediatric Bariatric Study Group. J Pediatr Surg.

[18] Nammi S, Koka S, Chinnala KM, Boini KM (2004). Obesity: an overview on its current perspectives and treatment options. Nutr J.

[19] Oberbach A, Neuhaus J, Inge T, Kirsch K, Schlichting N, Blüher S (2014). Bariatric surgery in severely obese adolescents improves major comorbidities including hyperuricemia. Metabolism.

[20] Olbers T, Beamish AJ, Gronowitz E, Flodmark CE, Dahlgren J, Bruze G (2017). Laparoscopic Roux-en-Y gastric bypass in adolescents with severe obesity (AMOS): a prospective, 5-year, Swedish nationwide study. Lancet Diabetes Endocrinol.

[21] Pedroso FE, Angriman F, Endo A, Dasenbrock H, Storino A, Castillo R (2018). Weight loss after bariatric surgery in obese adolescents: a systematic review and meta-analysis. Surg Obes Relat Dis.

[22] Pinheiro JA, Castro IRD, Ribeiro IB, Ferreira MVQ, Fireman PA, Madeiro MAD, Pontes ACP (2022). Repercussions of bariatric surgery on metabolic parameters: experience of 15-year follow-up in a hospital in Maceió, Brazil. Arq Bras Cir Dig.

[23] Pratt JSA, Browne A, Browne NT, Bruzoni M, Cohen M, Desai A (2018). ASMBS pediatric metabolic and bariatric surgery guidelines, 2018. Surg Obes Relat Dis.

[24] Rankin J, Matthews L, Cobley S, Han A, Sanders R, Wiltshire HD (2016). Psychological consequences of childhood obesity: psychiatric comorbidity and prevention. Adolesc Health Med Ther.

[25] Sueth DM, Tinoco ACA, Pompilho WM, Raeli JFFO, Zambrotti GTR, Antunes CM (2019). A cohort study on the outcomes of laparoscopic roux-en-y gastric bypass and laparoscopic sleeve gastrectomy regarding the change in body mass index, remission of comorbidities and quality of life after 12-month follow-up. SN Compr Clin Med.

[26] Van de Laar AW, van Rijswijk AS, Kakar H, Bruin SC (2018). Sensitivity and specificity of 50% excess weight loss (50%EWL) and twelve other bariatric criteria for weight loss success. Obes Surg.

[27] World Health Organization (2004). Global strategy on diet, physical activity and health, 2004.

[28] World Health Organization (2007). Growth reference data for 5-19 years n.d.

[29] Xanthakos SA (2009). Nutritional deficiencies in obesity and after bariatric surgery. Pediatr Clin North Am.

